# Determinants of high-sensitivity cardiac troponin T during acute exacerbation of chronic obstructive pulmonary disease: a prospective cohort study

**DOI:** 10.1186/1471-2466-12-22

**Published:** 2012-07-06

**Authors:** Arne Didrik Høiseth, Torbjørn Omland, Tor-Arne Hagve, Pål H Brekke, Vidar Søyseth

**Affiliations:** 1 Dept. of Medicine, Akershus University Hospital and Institute of Clinical Medicine, Akershus University Hospital, University of Oslo, Oslo, Norway; 2 Unit of Medical Biochemistry, Division of Diagnostics and Technology, Akershus University Hospital and University of Oslo, Oslo, Norway

## Abstract

**Background:**

A high-sensitivity cardiac troponin T (hs-cTnT) concentration above the 99^th^ percentile (i.e. 14 ng/L) is common during Acute Exacerbation of Chronic Obstructive Pulmonary Disease (AECOPD) and associated with increased mortality. The objective of the study was to identify factors associated with hs-cTnT levels during AECOPD.

**Methods:**

We included 99 patients with AECOPD on admission. As 41 patients had one or more repeat admissions, there were 202 observations in the final analysis. We recorded clinical and biochemical data, medication, spirometry, chest radiographs, and ECGs. The data were analysed for cross-sectional and longitudinal associations using ordinary least square as well as linear mixed models with the natural logarithm of hs-cTnT as the dependent variable.

**Results:**

Mean age at inclusion was 71.5 years, mean FEV_1_/FVC was 45%, and median hs-cTnT was 27.0 ng/L. In a multivariable model there was a 24% increase in hs-cTnT per 10 years increase in age (p < 0.0001), a 6% increase per 10 μmol/L increase in creatinine (p = 0.037), and a 2% increase per month after enrollment (p = 0.046). Similarly, the ratios of hs-cTnT between patients with and without tachycardia (heart rate ≥100/min) and with and without history of arterial hypertension were 1.25 (p = 0.042) and 1.44 (p = 0.034), respectively. We found no significant association between arterial hypoxemia and elevated hs-cTnT.

**Conclusion:**

Age, arterial hypertension, tachycardia, and serum creatinine are independently associated with the level of hs-cTnT on admission for AECOPD.

## Background

Cardiovascular disease (CVD) is frequent in chronic obstructive pulmonary disease (COPD) [1-5]. This is in part due to cigarette smoking being a strong common risk factor, but systemic effects of COPD are thought to independently promote CVD [6]. The role of systemic inflammation in the development of CVD is well established [7,8], and it is proposed that “systemic spill-over” from lung inflammation in COPD may explain the increased cardiovascular risk among these patients, both in general and particularly post exacerbation [6,9-12]. However, not only atherosclerotic heart disease, but also heart failure and arrhythmias are more common among COPD patients than in the general population [1-5].

We recently showed that myocardial injury, defined as high-sensitivity cardiac troponin T (hs-cTnT) above the 99^th^ percentile (i.e. 14 ng/L), was present in 74% of patients admitted for acute exacerbation of COPD (AECOPD) and that such injury carried a markedly increased risk of subsequent mortality [13]. We proposed four possible mechanisms, that may occur in concert, leading to elevated hs-cTnT during AECOPD: Type 1 and 2 myocardial infarctions (MI), increased right heart afterload due to either AECOPD alone or pulmonary embolism, and concomitant left heart failure. The determinants of troponin elevation in AECOPD are, however, sparsely studied.

In a previous cross-sectional study among patients hospitalised for AECOPD, we found that cardiac troponin T measured with a 4^th^ generation assay was positively associated with increasing serum creatinine, blood neutrophil cell count, and cardiac infarction injury score (CIIS), whereas it was negatively associated with hemoglobin level [14]. In that retrospective study, troponins were measured at the discretion of the attending physician in selected patients, which may have introduced a selection bias. In the present study we prospectively obtained data from patients hospitalised for AECOPD one or more times during the study period, thereby providing the opportunity to study the association between hs-cTnT and relevant covariables within patients, and reducing the influence of inter-individual differences.

Our objective was to identify clinical factors that are associated with the level of hs-cTnT in patients admitted with AECOPD using cross-sectional as well as longitudinal analyses of the association between these determinants and concurrent hs-cTnT.

## Methods

During 23 months in 2005 and 2006 we prospectively included 99 unselected patients admitted with AECOPD. Among these, 41 patients had data recorded on readmission during the inclusion period, and in total we gathered data on 219 admissions. On each admission we recorded heart rate (HR), blood pressure (BP), body temperature, respiratory rate, arterial blood gas (pH, PaCO_2_, PaO_2_), arterial oxygen saturation (SaO_2_), use of accessory respiratory muscles, wheezing, and chest pain. Mean arterial pressure (MAP) was estimated by the formula MAP = 1/3*systolic BP + 2/3*diastolic BP.

Serum and plasma from blood drawn on admission were stored at −80 °C for subsequent analysis of creatinine and hs-cTnT (cobas e 411 immunoanalyser, Roche diagnostics). According to the manufacturer of the hs-cTnT assay, the lower limit of detection is 3.0 ng/L, and the 99^th^ percentile in healthy volunteers was 14 ng/L. The lowest hs-cTnT level with 10% coefficient of variation was 13 ng/L. Glomerular filtration rate (GFR) was estimated by MDRD and Cockcroft-Gault formulae [15,16]. From the hospital records we recorded hemoglobin (Hb), leucocytes with neutrophil count, platelets, electrolytes, and C-reactive protein (CRP). Chest radiographs were examined by two physicians blinded for clinical data. Presence or absence of cephalisation, pneumonic infiltrates and pleural effusion in addition to the size of the heart and thoracic cavity in the frontal plane were recorded. ECGs recorded on admission were scored using CIIS. A score ≥20 has been shown to be a good indicator of prior MI [17], and to be associated with increased mortality in AECOPD patients [18]. Two physicians independently scored each ECG. When they disagreed on whether the CIIS was above or below 20, the score of a third physician was used. All three investigators were blinded to other data. ECGs were also analysed for the presence of arrhythmia, bundle branch block, left ventricular hypertrophy (LVH, assessed by Sokolow-Lyon criteria), signs of prior MI, or acute ischemia. We considered pathological Q-waves, loss of R-waves, T-wave inversion, and left bundle branch block to be signs of prior MI. ST-segment elevation or depression were recorded as signs of acute ischemia unless it was considered to be secondary to LVH.

Spirometry during stable phase was recorded when available. When several measurements were done, post bronchodilatation measurements prior to inclusion were preferred. Body mass index (BMI) was calculated from weight and height as recorded on the spirometry report or from the hospital records when spirometry was missing. Medical history was obtained by patient interview and hospital records. Patients were categorised as current, former (>1 year abstinence) or never smokers. Further details regarding patient inclusion and data gathering are described in a previous paper [13].

The study was approved by the Data Inspectorate and reviewed by the Regional Committee for Research Ethics. All included patients provided written informed consent to the participation in the study.

### Statistical analysis

Due to the skewed distribution of hs-cTnT, the natural logarithm of hs-cTnT (lnTnT) was used as the dependent variable in the analyses. Samples with hs-cTnT below the limit of detection (i.e. 3.0 ng/L), were assigned a value of 3.0. Outliers were identified by visual inspection of the data points. Individual assessment of outliers determined whether they were to be excluded from further analyses. The analyses were performed in four steps: First, we analysed cross-sectional associations between lnTnT and variables recorded on the index admission using Student *t*-test. The continuous variables were dichotomised at predifined cut-offs: Age at the mean, FEV_1_/FVC at the lower quartile, BMI at the lower limit of normal (i.e. 20 kg/m^2^), HR at 100/min, MAP at 90 mmHg, creatinine and neutrophil count at the upper quartile, pH at 7.30, PaCO_2_ at 6.3 kPa, PaO_2_ at 7.0 kPa, Hb at 12 g/dL in women and 13 g/dL in men, CRP at 50 mg/L, and CIIS at 20 points. In addition, the association between lnTnT and the following categorical variables were analysed: Gender, smoking status, history of coronary artery disease, heart failure, arterial hypertension, atrial fibrillation or diabetes, use of beta blockers, diuretics, ACE-inhibitors (ACEI) or angiotensin-II receptor blockers (ARB), statins, acetylic salicylic acid, or Warfarin, presence of atrial fibrillation, LVH, MI, or ischemia on ECG, peripheral edema, chest pain, and infiltrate or cephalisation on chest radiograph. Associations between continuous covariables at baseline and lnTnT were also analysed in a univariable linear regression model.

Second, in patients with repeat admissions, we investigated the level of lnTnT over time; first graphically and then by using time and time squared as independent variables in a linear mixed model (LMM) [19].

In the third step, we investigated intra-individual univariable associations between lnTnT and each of the continuous covariables. We identified the minimum and maximum values of continuous time-dependent variables along with the corresponding values of hs-cTnT. We then analysed the univariable associations between lnTnT and each of the continuous covariables using LMM with random intercept. From these analyses, the antilogarithm exp(β) of the coefficient (β) between lnTnT and each covariate can be interpreted as the relative change in lnTnT for a given change in the covariate.

Fourth, the variables that were associated with lnTnT with a p-value <0.2 in the cross-sectional or longitudinal analysis were included in the initial multivariable LMM. In this model we investigated candidate covariance structures and a model with random intercept. The models were compared using the Akaike Information Criteria (AIC). Using the model with the lowest AIC, we then manually reduced the model by backward elimination of variables with p-values <0.05 unless their removal increased the AIC statistic. Finally, we investigated the changes made by adding survival status and neutrophil count to the final model. Gender was kept in the model by convention.

All analyses were performed in SAS 9.2 (SAS Institute Inc., Cary, NC, USA), using PROC MIXED for the LMM.

## Results

### Cross sectional analyses at baseline

**Figure 1:**
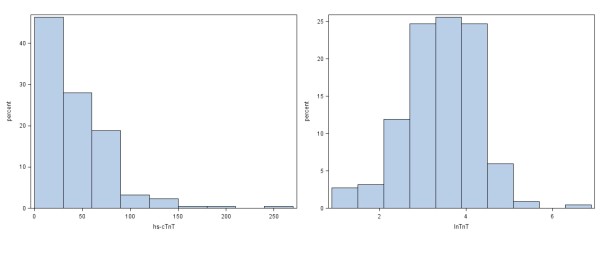
**Distribution of high-sensitivity cardiac troponin T.** The distribution of high-sensitivity cardiac troponin T (hs-cTnT, left panel) and their natural logarithms (lnTnT, right panel) in 219 admissions among 99 patients with acute exacerbation of COPD. In the left panel, an outlayer (hs-cTnT = 609 ng/L) has been removed.

Mean age at inclusion was 71.5 years (standard deviation (SD) 9.0), mean FEV_1_/FVC was 45% (SD 0.14), and median hs-cTnT was 27.0 ng/L (interquartile range (IQR) 13.4–51.0). LnTnT was close to normally distributed with mean 3.24 (SD 0.97), corresponding to a geometric mean of hs-cTnT = 25.6 ng/L. Of the 219 samples, two (0.9%) had hs-cTnT below the detection limit. The distribution of hs-cTnT and lnTnT are shown in Figures 1A and 1B. An outlier (hs-cTnT = 609 ng/L) was an 82 year old male in GOLD stage II with a history of arterial hypertension and heart failure who was admitted with COPD symptoms and treated accordingly. He is excluded from Figure 1A, but is included in Figure 1B and the analyses.

Using the Cockcroft-Gault formula, mean GFR was 85 mL/min (SD 31). Using the MDRD formula, 15.5% of the 219 samples had estimated GFR <60 mL/min/1.73 m^2^, and 2% were <30 mL/min/1.73 m^2^.

Spirometry was available in 88 patients. 80 of these (91%) were post bronchodilatation measurements. BMI was unavailable in five patients, and arterial blood gas was missing in eight. In total, complete datasets were available for 202 admissions. The results of the cross sectional univariable associations with hs-cTnT are shown in Table 1 for those variables reaching a level of significance of p <0.2. There were no significant associations between neutrophil count or CIIS ≥20 (*t*-test p-values 0.692 and 0.367, respectively). Moreover, we did not find any association between hs-cTnT and lung function. The patients with FEV_1_/FVC in the lower quartile (<0.34%) had a geometric mean of hs-cTnT = 26.1 ng/L compared to 26.6 ng/L among patients with FEV1/FVC in the upper three quartiles (p = 0.945). When restricting the analysis to the 80 patients with post bronchodilatation measurements, the p-value was 0.890. There was no statistically significant difference in hs-cTnT level among patients who had spirometry recorded and those who had not (geometric mean of 26.4 ng/L and 20.9 ng/L, respectively, p = 0.438).

**Table 1:** **Geometric mean of high-sensitivity cardiac Troponin T (hs-cTnT) by covariables associated with lnTnT (Student*t*-test p<0.20)**

	**Geometric mean of hs-cTnT, ng/L**		
	**(number of patients)**		
**Covariable**	**No**	**Yes**	***p*-value**
**Demographics and medical history**			
Age >71.5 years *	16.9 (50)	38.9 (49)	<0.0001
Female	29.1 (52)	22.2 (47)	0.153
Coronary artery disease	28.8 (72)	18.9 (27)	0.058
Arterial hypertension	21.8 (68)	37.0 (31)	0.011
Current or recent smoker	37.4 (48)	22.2 (51)	0.166
BMI <20 kg/m^2^	22.9 (62)	33.1 (32)	0.083
**Clinical findings**			
Chest pain	26.8 (89)	16.4 (10)	0.130
Heart rate ≥100/minute	28.2 (49)	23.3 (50)	0.052
**Laboratory tests**			
Low hemoglobin ^†^	23.1 (76)	36.6 (23)	0.045
Creatinine >86 μg/L^‡^	22.4 (69)	34.8 (30)	0.038
**Chest radiograph**			
Cephalisation	23.8 (83)	37.0 (16)	0.096
**Medication on admission**			
Diuretic (loop or thiazid)	22.6 (73)	36.2 (26)	0.033
ACEI or ARB	23.6 (76)	33.8 (23)	0.124
Statin	27.9 (79)	18.0 (20)	0.070

The table is based on each patient’s index admission only.

*Corresponds to the mean age; ^†^Defined as Hb <12 g/L in women and <13 g/L in men; ^‡^Corresponds to the upper quartile; *ACEI*, angiotensin converting enzyme-inhibitor; *ARB*, angiotensin receptor blocker.

### Longitudinal analyses

41 patients had one or more (maximum 10) repeat admissions, adding up to a total of 161 readmissions. Table 2 shows the intraindividual variation in continuous time-dependent covariables and the corresponding hs-cTnT values. It indicates a considerable intra-individual variation of the covariables. However, as shown in Table 3 (two right columns), univariable analyses revealed that only creatinine, PaO_2_, and heart rate (borderline) were significantly associated with lnTnT.

**Table 2:** The median of the minimum and maximum values of selected continuous time-dependant covariables and the corresponding values of high-sensitivity cardiac Troponin T in patients with repeat admissions for Acute Exacerbation of COPD

	Covariable (C)		Corresponding hs-cTnT (ng/L)	
	Minimum,	Maximum,	At minimum C,	At maximum C,
	median (IQR)	median (IQR)	median (IQR)	median (IQR)
MAP, mmHg	82.0 (71.7 – 93.7)	108.3 (92.0 – 126.7)	33.7 (18.8 – 60.8)	37.8 (18.3 – 72.4)
Creatinine, μmol/L	59 (47 – 67)	74 (60 – 91)	33.0 (19.6 – 60.3)	44.6 (21.2 – 77.4)
CRP, mg/L	10 (<5 – 20)	76 (26 – 141)	37.8 (19.2 – 72.4)	33.0 (22.0 – 60.3)
Neutrophil count, 10^9^/mL	5.6 (4.2 – 7.7)	11.8 (9.0 – 16.7)	34.2 (18.8 – 66.7)	32.4 (21.9 – 60.3)
Hemoglobin, g/L	12.6 (11.5 – 13.9)	14.0 (13.0 – 15.3)	32.4 (19.6 – 53.1)	36.7 (19.2 – 64.4)
Heart rate, /min	88 (75 – 99)	117 (101 – 126)	33.9 (18.8 – 54.0)	37.8 (21.8 – 68.4)
PaCO_2_, kPa*	5.32 (5.0 – 6.4)	6.9 (5.9 – 8.4)	33.0 (18.3 – 60.3)	37.8 (25.6 – 63.0)
PaO_2_, kPa*	6.7 (6.0 – 8.1)	9.8 (8.7 – 10.9)	37.5 (22.4 – 77.4)	31.4 (18.1 – 61.0)

The table is based on 161 admissions in 41 patients.

* Missing in eight admissions. *Hs-cTnT*, high-sensitivity cardiac troponin T; *IQR*, interquartile range; *MAP*, Mean arterial pressure; *CRP*, C-reactive protein.

**Table 3:** Univariable associations, expressed as the antilogarithm (exp(β)) of the coefficient β, between continuous time dependent covariables and the natural logarithm of hs-cTnT (lnTnT) assessed using an ordinary least square linear regression model (OLS, index admission) and a linear mixed model (LMM, repeat admissions)

		Interindividual associations between covariables and lnTnT (OLS)		Intraindividual associations between covariables and lnTnT (LMM)	
		Index admission in 99 patients		161 repeat admissions in 41 patients	
Covariable	Change in covariable	exp(β) (95% CI)	***p*-value**	exp(β) (95% CI)	***p*-value**
MAP	+5 mmHg	1.002 (0.951 – 1.06)	0.944	1.003 (0.98 – 1.03)	0.792
Creatinine	+10 μmol/L	1.08 (1.03 – 1.14)	0.004	1.10 (1.04 – 1.16)	0.0004
CRP	+10 mg/L	1.01 (0.97 – 1.05)	0.680	1.005 (0.99 – 1.02)	0.478
Neutrophil count	+5x10^9^/mL	0.93 (0.75 – 1.17)	0.555	1.005 (0.99 – 1.02)	0.468
Hemoglobin	+1 g/L	0.96 (0.85 – 1.08)	0.477	1.03 (0.68 – 1.6)	0.902
Heart rate	+5 bpm	0.98 (0.94 – 1.02)	0.392	1.02 (0.998 – 1.04)	0.078
PaCO_2_*	+1 kPa	1.09 (0.96 – 1.24)	0.195	1.05 (0.98 – 1.12)	0.144
PaO_2_*	+1 kPa	0.97 (0.87 – 1.08)	0.573	0.96 (0.94 – 0.99)	0.008

*Missing in eight admissions. *OLS*, ordinary least square linear regression model; *LMM*, linear mixed model; *MAP*, Mean arterial pressure; bpm, beats per minute.

According to the criteria outlined above, the following variables were included in the initial multivariable model: age, gender, time since inclusion, history of coronary artery disease or arterial hypertension, smoking, BMI, chest pain, Hb, creatinine, cephalisation, use of diuretics, ACEI/ARBs or a statin, HR, PaCO_2_ and PaO_2_. In this model BMI, Hb, HR, PaCO_2_, and PaO_2_ were dichotomised as previously described. A model with fixed effects only gave the lowest AIC statistic, and a spatial exponential covariance structure had the best fit. Reduction of the full model gave the final model (model 1) shown in Table 4. Even though both hypoxemia (15% increase in hs-cTnT per kPa, p-value 0.086) and BMI <20 kg/m^2^ (28% increase, p-value 0.144) failed to reach significance, it is adjusted for in the final model as their removal increased the AIC statistic.

**Table 4:** Relative change in hs-cTnT (95% confidence interval) by changing covariables on admission for Acute Exacerbation of COPD

		Model 1		Model 2	
**Covariable**	**Change in covariable**	**Relative change in hs-cTnT**	***p*-value**	**Relative change in hs-cTnT**	***p*-value**
**Time independent**					
Age at inclusion	+5 years	1.24 (1.13 – 1.37)	<0.0001	1.19 (1.08 – 1.31)	0.0005
History of HT	Yes vs. no	1.44 (1.03 – 2.01)	0.034	1.29 (0.92 – 1.80)	0.138
**Time dependent**					
Heart rate ≥100/min	Yes vs. no	1.25 (1.01 – 1.54)	0.042	1.24 (1.02 – 1.52)	0.036
Creatinine	+10 μmol/L	1.05 (1.003 – 1.09)	0.037	1.04 (1.002 – 1.09)	0.037
Time since inclusion	+1 month	1.02 (1.0004 – 1.05)	0.046	1.02 (0.99 – 1.05)	0.051

Obtained from a linear mixed model using 202 admissions among 94 patients.

Model 1 is adjusted for gender, hypoxemia, and BMI <20 kg/m^2^. Model 2 is additionally adjusted for survival status. *hs-cTnT*, high-sensitivity cardiac troponin T; *HT*, arterial hypertension.

When survival status was added to the final model, it proved significantly associated with hs-cTnT, with a relative value of 1.58 (95% CI 1.11 – 2.23, p = 0.011) among individuals who died during follow-up, rendering model 2 in Table 4. The interaction term time*survival status was not statistically significant (p-value 0.095). When added to the final model, neutrophil count failed to reach significance both as a continuous variable (7% increase in hs-cTnT per 5 cells, p = 0.160) and dichotomised at the upper quartile (relative change of 15%, p = 0.085).

Removing the outlier from the analyses made no significant changes in any of the results above.

## Discussion

In this study hs-cTnT was positively associated with age, history of arterial hypertension, tachycardia, creatinine, and follow-up time among patients hospitalised for AECOPD. The strength of this study is that the patients were followed prospectively, and that as many as 41% of the patients were investigated at two or more admissions. Consequently, the associations could be investigated longitudinally as well as cross-sectionally. We have also used the novel highly sensitive cardiac troponin T assay, allowing us to measure concentrations down to about 1/10 of what was previously possible, finding detectable cTnT in almost all samples.

Regarding the association between hs-cTnT and arterial hypertension, one might speculate that this was due to the development of LVH, as LVH have been found to be associated with troponin in other studies [20,21]. However, there was no significant association between troponin and electrocardiographic signs of LVH in the present study. An alternative explanation may be the established role of arterial hypertension as a risk factor for the development of CVD.

The association between hs-cTnT level and creatinine deserves some comment. In clinical practice, we often experience elevated levels of troponin in patients with renal failure, but without overt cardiac disease. It has been previously debated whether this is due to reduced renal elimination of troponin, but it may rather be a result of subclinical release of cTnT in these patients, as renal and cardiac atherosclerotic disease are pathophysiologically similar and partly overlapping. It may be worth noting that the majority of our patients had normal creatinine, an observation that favors the latter of the two theories. It may be argued that due to reduced muscle mass, patients with advanced COPD may have decreased renal function in spite of creatinine within the reference range. The finding that the addition of low BMI improves the fit of the final model, adds to this argument. We therefore estimated GFR, but still found that renal function was preserved in the vast majority of patients.

Although the cross-sectional analyses of the baseline data indicated an inverse association between tachycardia and hs-cTnT, longitudinal multivariable analysis showed a significant positive association between elevated hs-cTnT and tachycardia. This may be due to an effect of heart rate per se, but it may also reflect worsening of pulmonary hypertension and increased myocardial strain, undiagnosed pulmonary embolism, or a type 2 MI caused by insufficient oxygen delivery relative to the increased demand during tachycardia. Along this line of thinking, we would also expect an association between reduced PaO_2_ and hs-cTnT. Although we found such an association in the univariable longitudinal analysis, it was not significant in the cross sectional or multivariable analyses. Hence, our data do not support that elevated hs-cTnT in these patients can be explained as a type 2 MI mediated hypoxemia. However, patients with normal arterial oxygen tension in the emergency room may have received oxygen therapy in the ambulance, yet have had severe arterial hypoxemia before admission to the hospital causing cTnT leakage from the cardiomyocytes. Thus, the lack of association between hypoxemia and elevated hs-cTnT should be interpreted with caution.

Our analyses are based on data from repeat admissions by applying the linear mixed model [19]. Among the strengths of the model is that it does not require the outcome variable to be independent between observations, so one can use repeat observations from the same individual. Moreover, by using an appropriate covariance structure, the model allows us to have unbalanced data with different number of observations per patient and different timing of these. Thus, the analyses are based not only on inter-individual differences, but also on intra-individual changes in covariables over time, effectively allowing patients with repeat admissions to serve as their own controls. Hence, the association between hs-cTnT and covariables (such as oxygen tension and creatinine changes) can be investigated intra-individually. Comparison of the results of these univariable analyses show that the associations with creatinine were highly significant in the cross-sectional as well as in the longitudinal models with the estimates being nearly identical. There is discrepancy regarding heart rate, with p-value <0.10 in the LMM only. Although the design of the study does not permit any conclusions to be drawn regarding cause and effect, this discrepancy may suggest that there is an association between tachycardia and cTnT release in susceptible individuals.

Our group has previously published results from a retrospective cohort study of 441 patients with AECOPD, identifying creatinine, hemoglobin, neutrophil count, heart rate and CIIS as independent predictors of elevated cTnT (≥0.04 μg/L) [14]. In the present prospective study, we confirm that age, creatinine, and heart rate are independently associated with hs-cTnT level. In addition, we observed an association between hs-cTnT and history of arterial hypertension. CIIS and neutrophil count were not significantly associated in the present analyses, perhaps due to the relatively modest sample size. On the other hand, considerable intra-individual variation of neutrophil count was observed, thereby weakening the hypothesis of a relationship between neutrophils and cTnT. Other markers of inflammation than leucocytes, fibrinogen in particular, have been shown to be associated with COPD, its severity and exacerbation frequency [22-27]. Fibrinogen was not measured in this study, but one might speculate whether this inflammatory marker might be more closely associated with cTnT, as it may be a risk factor for the development of CVD.

Other limitations of this study include the modest size of the cohort, which may explain the inconsistency regarding tachycardia, and the nonsignificant association with hypoxemia. Nevertheless, we identify associations that are both statistically significant and physiologically plausible. The strengths of the associations may be unimpressive, with an estimated 44% increase in hs-cTnT in patients with hypertension as the strongest association. The range of hs-cTnT concentrations is small, however, so strong associations can not be expected. Moreover, in spite of this narrow range, relatively small changes in hs-cTnT are associated with markedly adverse prognosis in this group [13]. Thus, it is important to identify determinants of cTnT elevation in AECOPD.

Although patients with confirmed PE were excluded from the study, the diagnosis was not systematically investigated, and unrecognised PE may have influenced the results.

## Conclusion

Age, arterial hypertension, tachycardia, and serum creatinine are independently associated with the level of hs-cTnT at admission for AECOPD.

## Competing interests

The authors declare that they have no competing interests.

## Authors’ contribution

All authors had full access to the original data and vouch for the completeness and veracity of the data and data analyses. All authors contributed to data interpretation and to the writing of the report, made final decisions on all parts of the report, and read and approved the final manuscript. PHB, TAH, TO and VS designed the study. PHB interviewed and enrolled the patients. AHD and VS confirmed the diagnoses, undertook the statistical analyses and generated tables and figures. AHD and PHB analysed the ECGs. VS reviewed the radiographs.

## Pre-publication history

The pre-publication history for this paper can be accessed here:

http://www.biomedcentral.com/1471-2466/12/22/prepub
